# Characterization of Culturable Plant Growth-promoting Bacteria Isolated from *Amaranthus hypochondriacus* L. Roots

**DOI:** 10.1264/jsme2.ME26025

**Published:** 2026-09-18

**Authors:** Fátima Alejandra Talamantes-Herrera, Alberto Barrera-Pacheco, Eduardo Espitia-Rangel, Ana Paulina Barba-de la Rosa

**Affiliations:** 1 IPICyT, Instituto Potosino de Investigación Científica y Tecnológica, (División de Biología Molecular), Camino a la Presa San José No. 2055, Lomas 4ª sección, San Luis Potosí, (S.L.P.), 78216 Mexico; 2 INIFAP, Instituto Nacional de Investigaciones Forestales, Agrícolas y Pecuarias, Campo Experimental Valle de México, Coatlinchan, Texcoco, Estado de Mexico, 56250 Mexico

**Keywords:** amaranth, bioinoculants, diazotrophs, total indole, iron solubilization, phosphate solubilization

## Abstract

Amaranths plants grow under several abiotic stress conditions and produce high-quality edible seeds. Despite their high agronomic and nutritional potential, their endophytic bacteria have not yet been characterized. This study aimed to isolate *Amaranthus hypochondriacus* L. root endophytes and characterize their plant growth-promoting capacity. Twenty-six amaranth root putative diazotrophs were isolated and identified by 16S rRNA gene sequencing. Most of the isolated strains exhibited multiple plant growth-promoting traits, such as iron and phosphate solubilization and the capacity to form biofilms. Total indole production by one of the isolated bacteria, identified as *Pantoea vagans*, was high (124.6 μg mL^–1^). Four endophytes that showed coculture compatibility, identified as *Lelliottia*, *Pantoea vagans*, *Pseudomonas cf. fluorescens*, and *Pseudomonas rhodesiae*, were selected for their individual or combined (consortium) inoculation in the model plant *Arabidopsis thaliana*. The consortium resulted in significant increases in fresh and dry biomass accumulation in *Arabidopsis* plantlets. These results highlight the diversity of amaranth root endophytes and their potential as biofertilizers. Further research to verify the consortium in amaranths and other commercial crops under greenhouse and field conditions is warranted.

Climate change and population growth demand an increase in food supply, which necessitates the greater use of pesticides and chemical fertilizers. Several alternatives for sustainable food production have been proposed, including the use of underutilized crops (Alotaibi, 2023). Amaranths (*Amaranthus* spp.) are ancient crops that thrive in disadvantaged conditions under stress settings, including saline, acid or alkaline soils, drought, and high temperatures (Vargas-Ortiz *et al.*, 2021). Furthermore, amaranth crops produce edible seeds with high quality protein, which in addition to their good balance of essential amino acids also contains encrypted peptides with demonstrated antihypertensive, antidiabetic, and antioxidant effects. Amaranth seeds are a gluten-free food and rich source of essential minerals and phytochemicals (Barba de la Rosa *et al.*, 2009). Several species of amaranths, including *Amaranthus cruentus*, *A. hypochondriacus*, and *A. hybridus*, have the ability to sequester heavy metals and, thus, have potential in phytoremediation (Hunková *et al.*, 2024). However, despite the positive agronomic and nutritional qualities of amaranth plants and seeds, their associated microorganisms have not yet been characterized.

Endophytic bacteria are environmentally friendly microbial agents that colonize inside plant tissues without causing damage and play important roles in plant health. Endophytes have been shown to enhance plant development, function as biological control agents, and exhibit resilience to a wide array of environmental stresses, including heavy metal stress (Compant *et al.*, 2021; Ma *et al.*, 2024). Several endophytes are regarded as plant growth-promoting bacteria (PGPB), and associate with plants through direct or indirect mechanisms. One of the direct mechanisms is the metabolism of essential nutrients needed by plants, mainly nitrogen (N), phosphate (P), and iron (Fe). PGPB also synthesize one or more phytohormones or their precursors, such as auxins, cytokinins, and gibberellins. Indirect mechanisms are those related to protection against pathogens by antagonism, antibiotic production, nutrient competition, root colonization or production, and the release of enzymes, such as glucanases and chitinases (Lugtenberg and Kamilova, 2009). Since endophytes are important microbial resources for agricultural applications, there is a growing interest in identifying PGPB in underexplored environments, as well as endophytes from underutilized crops, and harnessing their properties to develop alternative strategies for improving resilience and promoting the growth of commercial crops (Chaudhary *et al.*, 2022; Aishwarya *et al.*, 2025). Chinampas are traditional Mesoamerican agroecosystems that consist of islands built from lake sediments and organic matter, and are maintained under constant moisture and high fertility without synthetic fertilizers (Ebel, 2020; Pérez-Belmont *et al.*, 2021). The microbial communities adapted to these conditions may harbor unique plant growth-promoting traits, such as enhanced nutrient cycling, stress tolerance, and compatibility with wetland crops. Therefore, the isolation of endophytes from amaranths grown in chinampas offers the potential to discover novel bioinoculants adapted to sustainable, low-input agricultural systems (Pérez-Belmont *et al.*, 2021). While bacteria in the rhizosphere of amaranth plants have been‍ ‍investigated, the endophytic community of *A. hypochondriacus* roots, particularly those cultivated in the chinampas agroecosystem, has yet to be exami­ned in detail. Therefore, the present study aimed to isolate root endophytes from *A. hypochondriacus* L., which was cultivated in this specific niche. Amaranth root endophytes were isolated using culture media methods and identified by 16S rRNA gene sequencing. The PGBP traits of isolates were evaluated *in vitro* (iron and phosphate solubilization, total indole production, and biofilm formation). Isolates were tested *in vivo* for their potential as bioinoculants using *Arabidopsis thaliana* plantlets. The results obtained herein represent the first investigation of amaranth root endophytes, which have a high agronomic potential to contribute to improvements in agricultural practices.

## Materials and Methods

### Collection of plant material

Seeds of *A. hypochondriacus* L. race Azteca were germinated in chinampas. Seedlings were transplanted to Tehutli mountain (Fig. 1), Santiago Tulyehualco, Xochimilco Delegation, México (19° 13′ 50″ N; 99° 1′ 32″). The roots of 27 plants were harvested at flowering and transferred on ice. Root samples (3 g) were transferred into sterile tubes with 35 mL of Buffer-R (50 mM KH_2_PO_4_, 50 mM K_2_HPO_4_, and 0.1% Triton X-100). Rhizosphere and endosphere fractions were processed according to a previously reported protocol (Coleman-Derr *et al.*, 2016). Briefly, root samples were ultrasonicated for 10 min to eliminate adhered microorganisms and then superficially sterilized by washing in 10% hypochlorite solution, 70% ethanol, phosphate-buffered saline (PBS) pH 7.4, and sterile Milli-Q water. As a negative control, the final wash was inoculated in a rich medium to remove microorganisms.

### Isolation of culturable putative diazotrophic amaranth root endophytes

Root samples were ground, serial diluted, and plated on N-free Jensen’s medium (20 g L^–1^ sucrose, 1.0 g L^–1^ K_2_HPO_4_, 0.5 g L^–1^ MgSO_4_.7H_2_O, 0.5 g L^–1^ NaCl, 0.1 g L^–1^ FeSO_4_, 50 mg L^–1^ Na_2_MoO_4_, 2.0 g L^–1^ CaCO_3_, and 15 g L^–1^ agar, pH 7.0). Plates were incubated at 30°C for 7 days, visible colonies were purified, named AhX# (Ah for *A. hypochondriacus*, X for Xochimilco, and # the number assigned to the isolate), and stored in glycerol at –20°C. Assays were performed in triplicate for all colonies collected.

### Molecular identification of bacterial endophytes

DNA was extracted from each bacterial isolate using a CTAB-based protocol (Mesapogu *et al.*, 2013). Quality control was conducted using Nanodrop 8000 (ThermoFisher Scientific). DNA integrity was exami­ned using a 1% agarose gel. In 16S rRNA gene sequencing, the 27F (5′-AGRGTTYGATYMTGGCTCAG-3′) and 1492R (5′-RGYTACCTTGTTACGACTT-3′) primers were used. The PacBio Sequel II platform was employed (Pacific Biosciences) for long amplicons (20,000 reads sample^–1^). The IMR service was used (Dalhousie University, Nova Scotia, CAN). Reads were processed using DADA2 v1.28 (Callahan *et al.*, 2016) for filtering, trimming, the removal of chimeras, and single tones. Amplicon sequence variants (ASVs) were clustered using 100% identities by the SILVA *v*138.1 database (Quast *et al.*, 2013). A phylogenetic anal­ysis was conducted in MEGA X using the Maximum Likelihood (ML) method with the Find Best DNA Model. Based on the Bayesian Information Criterion, the best-fit model was HKY+G+I (Hasegawa-Kishino-Yano with Gamma-distributed rates and the percentage of invariant sites). We then constructed the ML tree using this model with 2,000 bootstrap replicates to assess branch support. Gaps were treated using partial deletions with a 95% site coverage cut-off to retain as much informative data as possible. The Nearest-Neighbor-Interchange (NNI) heuristic method was used for the tree search (Kumar *et al.*, 2018). The anal­ysis included the 26 sequences corresponding to amaranth root endophytes plus 19 reference strain sequences with NCBI accession IDs, and two *Legionella* sequences added as an outgroup (NCBI accession ID: NR 104921.1 and NR 152697.1). Regarding the isolates that did not cluster directly with a type strain with strong support, the designation “*cf.*” (*confer*, meaning “compare with”) was used to indicate a tentative species assignment, while “*sp.*” was used when no confident species-level identification was made. This conservative approach acknowledged the limited resolution of the 16S rRNA gene for discriminating closely related species within the genus *Pseudomonas*.

### Screening of plant growth-promoting traits of amaranth root endophytes

#### Iron- and Phosphate-solubilizing indices

The iron solubility by amaranth root endophytes was assessed in CAS-agar media (Srimathi and Ann Suji, 2018). An aliquot (3.5 μL) of bacteria grown at an optical density at 600 nm (OD_600_) of 0.5 on LB broth was streaked on plates containing Chromo Azurol S (Sigma Aldrich) and incubated at 28°C for 2 days. The halo of mineral solubilization was measured after 48 h and the siderophore production index (SPI) was calculated using equation (1).

$$\text{SPI or PS}=\frac{\left(\text{Solubilization zone diameter}+\text{Colony diameter}\right)}{\left(\text{colony diameter}\right)}\;(1)$$

To measure the phosphate-solubilizing index (PSI), bacteria were inoculated on plates of Pikovskaya’s Agar (PKV) (10 g L^–1^ glucose, 0.5 g L^–1^ yeast extract, 5.0 g L^–1^ Ca_3_(PO_4_)_2_, 0.5 g L^–1^ (NH_4_)2SO_4_, 0.2 g L^–1^ NaCl, 0.1 g L^–1^ MgSO_4_.7H_2_O, 0.1 g L^–1^ KCl, 1 mg L^–1^ MnSO_4_, 1 mg L^–1^ FeSO_4_, and agar 15 g L^–1^, pH 7.0) and incubated at 28°C for 2 days. PSI was calculated using equation (1).

### Total indole production and biofilm formation capacity

Indole-producing bacteria (indole acetic acid [IAA] equivalent) were screened using Salkowski’s reagent (SR) as described by Gang *et al.* (2019). Briefly, isolated bacteria were inoculated in NB medium (1 g L^–1^ D-glucose, 15 g L^–1^ peptone, 6 g L^–1^ NaCl, and 3 g L^–1^ yeast extract, pH 7.5±0.2) and then incubated at 30°C overnight at 120 rpm. A fresh culture (100 μL) was prepared to inoculate test media (NB+0.15% L-Trp [w/v]) and then incubated at 30°C at 120 rpm for 7 days in the dark. Reads at 536 nm were taken every 24 h using a Multiscan GO microplate spectrophotometer (ThermoFisher Scientific) using 1 mL of the upper layer and mixing with RS (1:1). NB medium was used as the blank. A standard curve of IAA from 0.1–100 μg mL^–1^ was used as a reference. Biofilm formation was assessed using a colorimetric stain protocol (Erriu *et al.*, 2012). Overnight cultures of YEP broth (20‍ ‍g‍ ‍L^–1^ peptone and 10 g L^–1^ yeast extract, pH 7.5±0.2) were incubated at 28±2°C for 24 h until OD_600_ reached 1.0. Dilutions of 1:100 were made and aliquots (200 μL) were placed in 96-well plates, which were then incubated at 28°C for 24 h. After 24 h, the wells were washed with PBS (pH 7.4), stained with crystal violet (CV) 0.4%, and then washed again. CV was solubilized in 33% acetic acid solution. OD_600_ was measured using a Multiscan GO (ThermoFisher). YEP broth was used as the blank. Five replicates were made for each isolate and each assay was performed in triplicate. Results were reported as the OD of the bacterial biofilm versus its weight (Stepanović *et al.*, 2000).

### Abiotic stress tolerance of isolated endophytes

The effects of osmotic tolerance (drought tolerance) on bacterial growth were assessed in overnight cultures in LB media containing polyethylene glycol (PEG) 8000 (Sigma-Aldrich). Bacterial cultures were diluted 1:100 and 3.5 μL was inoculated into osmotic stress LB media (Ψ=–3.5 and –7.5 bar) obtained according to equation (2) (Michel, 1983; Fiodor *et al.*, 2023).

$$\text{PEG}=\frac{\left(4-\sqrt{\left(5.16\;\Psi \;℃-560\Psi +16\right)}\right)}{\left(2.58℃-280\right)}\;(2)$$

where PEG is acquired PEG, Ψ is the desired hydric potential, and °C is the specific temperature.

Samples were incubated at 30°C for 7 days with constant agitation at 200 rpm. Culture growth was monitored at OD_600_. Fresh LB (Ψ=0 bar) was used as the control. All measurements were performed in triplicate.

### Isolate compatibility test and evaluation of plant growth-promoting isolates as bioinoculants

*In vitro* compatibility between preselected isolates was tested using the LB agar cross-streak diffusion test (Fiodor *et al.*, 2023). Fresh overnight cultures were diluted 1:100, streaked vertically on LB plates, and then incubated at 30°C for 24 to 72 h until colony growth. The second inoculum was a perpendicular streak and cultures were incubated again at 30°C for 24 to 72 h. Inhibition at the crossed line was subsequently recorded. *A. thaliana* ecotype Col-0 seeds were surface sterilized with a 20% (v/v) sodium hypochlorite solution for 10 min, washed three times with sterile distilled water, and then germinated on MS agar plates (Gilbert *et al.*, 2023) placed vertically in a growth chamber at 22°C under a 16 h light/8 h dark photoperiod with a light intensity of approximately 100 μmol m^–2^ s^–1^ and 60% relative humidity. After 4 days, seedlings were transplanted into fresh MS agar plates. Amaranth endophytes were grown on LB medium at 28°C for 24 h. On day 8, bacteria diluted 1:100 were streaked 1 cm away from the bottom of the *A. thaliana* Petri plate. At 15 days post-inoculation, plants were removed from MS plates. The fresh weight of seedlings was measured, they were then air-dried at 70°C for 72 h, and dry weight was measured. Each treatment consisted of 15 plants, and the experiment was repeated six times.

### Statistical anal­ysis

Statistical anal­yses were performed using GraphPad Prism v8.0.2. All data are reported as the mean±standard deviation (SD). Normality was assessed using the Shapiro-Wilk test. Regarding datasets with a normal distribution and for comparisons of multiple groups (iron and phosphate solubilization, total indole production, and osmotic stress tolerance), a one-way anal­ysis of variance (ANOVA) was used, followed by Tukey’s post-hoc test for multiple comparisons. The non-parametric Kruskal-Wallis test followed by Dunn’s post-hoc test were performed for datasets that did not follow a normal distribution (biofilm formation). Concerning *Arabidopsis* biomass data (fresh and dry weights), linear mixed-effects models were fit using the lme4 package in R (Bates *et al.*, 2025). Treatment (Control, AhX2, AhX3, AhX13, AhX15, and Com4+) was included as a fixed effect. Experimental replicate (six independent batches, each containing 15 plants per treatment) was included as a random intercept to account for plate-to-plate variations. Pairwise comparisons between treatments were performed with Tukey’s adjustment for multiple testing using the emmeans package. *P*<0.05 was considered to be significant for all tests.

## Results and Discussion

### Isolation and identification of amaranth bacterial endophytes

Amaranths are ancient crops, are regarded as one of the most nutritious plants on earth, and have been cultivated in chinampas since pre-Hispanic times (Espitia-Rangel *et al.*, 2022). However, the microbiota harbored by amaranth seedlings germinated in chinampas has yet to be investigated. In the present study, single colonies of 26 putative diazotrophic bacteria were isolated in Jensen’s medium. Isolated colonies were identified by 16S rRNA gene sequencing and purified (Table 1). A ML phylogenetic tree was constructed using the HKY+G+I substitution model with 2,000 bootstrap replicates, with *Legionella* sequences as the outgroup (Fig. 2). Branches with bootstrap support <50% are shown, but not discussed due to low confidence. The tree revealed two major clades. One clade comprised the family Enterobacteriaceae, including the genera *Lelliottia* (isolates AhX1, AhX2, AhX4, AhX16, and AhX26) and *Pantoea* (AhX3). *Lelliottia* isolates formed a well-supported group (bootstrap 99–100%) with the type strain *Lelliottia amnigena* JCM1237, confirming their genus level assignment. Isolate AhX3 clustered with high support (bootstrap 100%) with the *Pantoea agglomerans* type strain and was identified as *Pantoea vagans*. The second major clade corresponded to the genus *Pseudomonas*. Within this clade, several isolates formed well-supported groups with recognized type species. Isolates AhX11 and AhX12 clustered with *Pseudomonas mediterranea* G-229-21T (bootstrap 99%). Isolate AhX25 clustered with *P. azotoformans* NBRC 12693 (bootstrap 100%). Isolates AhX13, AhX14, AhX22, and AhX24 formed a cohesive group with the *P. rhodesiae* type strain CIP 104664 (bootstrap 85–97%). For difficult isolates, close relationships to described species were observed, but could not be unambiguously assigned due to limited phylogenetic resolution or because they did not cluster directly with the respective type strains, and thus, were given the provisional designation *Pseudomonas cf. kribbensis* AhX7 AhX8, AhX19, (bootstrap 92–100%). Isolates AhX9, AhX10, AhX15, AhX17, and AhX18 are reported as *Pseudomonas cf. fluorescens* (bootstrap 62–95%) and *Pseudomonas cf. koreensis* AhX20 (bootstrap 100%). Isolates AhX5, AhX6, AhX21, and AhX23 could not be assigned with confidence to any known species; they were reported as *Pseudomonas* sp. The outer group was represented by *Legionella spp.*, which was used for rooting the phylogenetic tree. Several *Pseudomonas* species have also been detected in the rhizosphere and rhizosheath of *A. hypochondriacus* (Moreno-Espíndola *et al.*, 2013, 2018), and *P. fluorescens* has been isolated from the root zone of *A. hybridus* (Oyeyiola, 2010). *P. fluorescens* has several traits that are useful for biocontrol; in addition to its N_2_-fixing capacity (based on growth in N-free medium), it has the ability to solubilize minerals that are absorbed by plants, it produces several bioactive metabolites, and may adapt to environmental stresses and promote plant growth and yield (Adnan *et al.*, 2017; Mehmood *et al.*, 2023). However, further studies, such as nifH gene identification and acetylene reduction assays, are needed to confirm nitrogenase activity.

### Characterization of plant growth-promoting activities of isolates

#### Iron and phosphate solubilization

Putative diazotrophic endophytic bacteria, which have the ability to solubilize iron and phosphate, are of vital importance in healthy plant development (Chaudhary *et al.*, 2022). Among the endophytes isolated in the present study, *Ah*X12 (*P. mediterranea*) exhibited the highest ability to chelate iron, activity that is related to siderophore production, with a SPI value of 5.7±0.1, followed by *Ah*X3 *Pantoea vagans* (4.1±0.0) and *Ah*X25 *P. azotoformans* (3.5±0.1) (Table 1). The PSI of the majority of the amaranth root endophytes isolated were high (Table 1). The highest PSI was observed for the two *P. rhodesiae*, *Ah*X14 (4.5±0.0) and *Ah*X22 (3.9±0.1), followed by *Ah*X15 or *P. cf. fluorescens* (3.8±0.2). Some bacteria with a high phosphate-solubilizing ability are *Pseudomonas*, *Bacillus*, *Pantoea*, *Burkholderia*, *Enterobacter*, and *Rhizobium* (Adnan *et al.*, 2017). Two *Lelliottia* strains were also identified in the present study. *Lelliottia* belongs to the *Erwiniaceae* family, which has not been reported before in amaranths. Several *Erwiniaceae* species have been used for agricultural purposes; *Erwiniacea* strain PD-1 isolated from mushroom compost was previously shown to produce high concentrations of indole-3-acetic acid (Kim *et al.*, 2024) and *Lelliottia sp.* JS-SCA-14 was found to possess iron- and phosphate-solubilizing traits (Jeon *et al.*, 2024). We also isolated one strain of *Pantoea vagans* (*Ah*X3, Table 1). *Pantoea* species are well known for their role in biocontrol, and there is increasing interest to isolate these species and elucidate the mechanisms by which they protect against pathogens (Duchateau *et al.*, 2024). *Pantoea* species have also been shown to promote nodulation and increase grain yield in chickpea plants (Tariq *et al.*, 2023).

### Quantitative indole acetic and biofilm production

IAA is the most common indole in the phytohormone group of auxins, and has been shown to regulate the growth and development of plants (Fu *et al.*, 2015). The total indole production capacity of isolated root endophytes was measured in this study. Among the isolates, AhX3 *Pantoea vagans* exhibited the highest indole production, reaching 124.6±0.29 μg mL^–1^ at 168 h. Notably, indole production by this isolate was still increasing at the final sampling point, indicating that maximum production had not yet been reached (Fig. 3). In contrast, *Ah*X2 Lelliottia *sp*. reached its maximum indole concentration of 18.9±0.05 μg mL^–1^ at 168 h, whereas *Ah*X21 *Pseudomonas* sp. attained its highest value of 15.83±0.14 μg mL^–1^ between 72 and 96 h (Fig. 3). Lower indole producers were *Ah*X25, *Ah*X15, and *Ah*X13 (*P. azotoformans*, *P. cf. fluorescens*, and *P. rhodesiae*, respectively) with values of 7.0 to 7.3 μg mL^–1^. IAA is a widely distributed intercellular signal molecule in microbial communities and is involved in multiple bacterial processes (Melander *et al.*, 2014). Indole production by *Klebsiella* was previously reported to peak at 215 μg mL^–1^ after 72 h (Gang *et al.*, 2019). Indole production by *Streptomyces fradiae* NKZ-259 reached a maximum at 82.36 μg mL^–1^ after 168 h (Myo *et al.*, 2019). Colonization by and the survival of *Pantoea vagans* strains in several plants appear to be mediated by phytohormone signaling induced by IAA production (Lara-Cortés *et al.*, 2019).

The formation of biofilms, which are composed of polysaccharides, proteins, and other molecules, is a key strategy used by bacteria to colonize plant roots (Mann and Wozniak, 2012). In the present study, biofilm formation was exami­ned using a well-established colorimetric protocol (Erriu *et al.*, 2012; Stepanović *et al.*, 2000). Our strongest biofilm-forming endophytes (*P. rhodesiae*, *P. cf. fluorescens*, and *P. azotoformans*) exhibited higher OD values, ranging from 0.65±0.15 to 2.24±0.3598 (Fig. 4) than those reported for *P. aeruginosa*, which showed an OD value of 0.38 and it is considered as a strong biofilm-forming model (Vital-Lopez *et al.*, 2015).

### Osmotic tolerance of amaranth root endophytes

Drought tolerance measured as osmotic stress was evaluated in liquid LB as the control (Ψ=0.0 bar) and in LB supplemented with PEG 8000 to obtain osmotic stress values of Ψ=–3.5 bar and –7.5 bar (Fig. 5). Bacteria have been shown to reduce osmotic stress by increasing host plant accumulation of osmolytes, such as proline, trehalose, and glycine betaine (Susilowati *et al.*, 2018). *Ah*X12 *P. mediterranea* exhibited the highest tolerance to osmotic stress. *Ah*X13 *P. rhodesiae* and *Ah*X15 *P. cf. fluorescens* exhibited growth increase until 5 days under both osmotic stress conditions. Different bacteria isolated from the soybean rhizosphere have been shown to grow under several osmotic stress conditions ranging from –1.0 to –2.0 MPa (Ashry *et al.*, 2022). A previous study demonstrated that IAA production by *Bacillus* DS4 strains was the highest (1.23 to 1.61 μg mL^–1^) under osmotic stress conditions of –0.15 to –1.2 MPa (Wang *et al.*, 2014). Furthermore, drought stress caused by a high PEG concentration in medium was found to stimulate auxin production by *Serratia sp.* and *P. stutzeri* (Hassen *et al.*, 2016). A higher yield of IAA (1.6 μg mL^–1^) by the highly tolerant *Bacillus sp.* E2 strain was obtained under an osmotic stress condition of –2.0 MPa (Ashry *et al.*, 2022). In the present study, *Ah*X2 *Lelliottia* produced 15 μg mL^–1^ of IAA, even under control conditions (Fig. 3), which was higher than previously reported values.

### Coculture compatibility of and bioinoculant test for amaranth root endophytes

The compatibility of isolated amaranth root endophytes was tested in cocultures. Of the 36 crosses conducted, four bacterial endophytes were selected based on their cross compatibility: *Ah*X2 *Lelliottia*, *Ah*X3 *Pantoea vagans*, *Ah*X13 *P. rhodesiae*, and *Ah*X15 *P.cf. fluorescens*. Each of these isolates, as well all of them in a consortium (Com 4+), were inoculated into *A. thaliana* plantlets. Significant changes were observed in leaf sizes and lateral roots between control plantlets and those treated with bacteria (Fig. 6A). The fresh and dry weights of *A. thaliana* plantlets were analyzed using linear mixed-effects models with treatment as the fixed factor and replicate as the random intercept. The mixed-effects anal­ysis of fresh weight (Fig. 6B) showed a significant effect of treatment (*P*<0.0001). Com4+ produced the highest estimated mean fresh weight (16.33 mg), which was significantly higher than those of all other treatments (*P*<0.0001 for each comparison). Mean fresh weights with AhX15 (9.95 mg) and AhX13 (9.67 mg) were significantly higher than those with AhX2, AhX3, and control (*P*<0.0001), but did not significantly differ from each other (*P*=0.9963). Mean fresh weights with AhX2 (5.35 mg) and AhX3 (4.72 mg) were not significantly different from each other (*P*=0.8752), but were both higher than that of the control (2.22 mg, *P*<0.0001). The mixed-effects anal­ysis of dry weight (Fig. 6C) also showed a significant effect of treatment (*P*<0.0001). Com4+ produced the highest estimated mean dry weight (1.176 g), which was significantly higher than those of all other treatments (*P*<0.05 for each comparison, including vs AhX15, *P*=0.0141). Mean dry weights with AhX15 (1.042 g) and AhX13 (0.987 g) were significantly higher than those with the control, AhX2, and AhX3 (*P*<0.0001), but did not significantly differ from each other (*P*=0.7546). Mean dry weights with AhX2 (0.595 g) and AhX3 (0.514 g) also did not significantly differ from each other (*P*=0.3542), but were both significantly higher than that of the control (0.186 g, *P*<0.0001). Biomass accumulation was the highest with Com4+, followed by the AhX15 and AhX13 treatments. The higher biomass observed with Com4+ than with some individual strains suggests a synergistic or additive effect among the four compatible isolates. However, the current experimental design cannot distinguish whether this was due to complementary PGP traits, such as one strain solubilizing phosphate and another producing indole compounds, or if the effect was primarily driven by a single highly effective strain within the consortium. Therefore, detailed pairwise combination experiments are needed to examine this interaction in more detail.

*P. rhodesiae* and *P. cf. fluorescens* alone significantly increased biomass production. *P. cf. fluorescens* has been shown to confer benefits to members of Brassicaceae, including *Arabidopsis*, by protecting them from biotic and abiotic stresses, promoting growth, and increasing yield (Rolón-Cárdenas *et al.*, 2021; Sah *et al.*, 2021). *Pseudomonas* play key roles in plant pathogen control and plant growth during their interactive organization with host plants (Fan *et al.*, 2020). A bacterial strain of *Lelliottia sp.* was recently shown to exert significant plant growth-promoting effects that were attributed to the phosphate-solubilizing ability of this strain (Jeon *et al.*, 2024). The attenuation of abiotic stress by bioinoculants is a common research field (Fan *et al.*, 2020; Sah *et al.*, 2021; Mehmood *et al.*, 2023). In the present study, the difference in biomass accumulation by *Arabidopsis* plantlets inoculated with AhX15 *P. cf. fluorescens* and AhX13 *P. rhodesiae* was noted in plants without any type of abiotic stress, showing alternative set-ups to induce plant growth. While the *Arabidopsis* model provided an efficient system for initial functional screening, these results are preliminary and based on a model plant system; therefore, they identify candidate strains for further evaluation rather than proving biofertilizer efficacy for amaranths. Further research is warranted to evaluate the biofertilizer efficacy of these isolates, particularly the consortium, on amaranths under controlled greenhouse and field conditions. This will be the crucial next step to establish whether they have potential as biofertilizers for this crop.

## Conclusion

Amaranth crops have been cultivated in chinampas, which are soils rich in biodiversity, since pre-Hispanic times. Amaranth roots harbor various bacteria, and 26 were isolated using culture-dependent techniques in the present study. Four bacteria, *P. rhodesiae, P. cf. fluorescens*, *Lelliottia*, and *Pantoea vagans*, showed PGPB traits, such as iron and phosphate solubilization and indole production, and exhibited coculture compatibility. These bacteria, when inoculated in the model plant *A. thaliana*, resulted in the higher accumulation of fresh and dry biomasses. Further studies are‍ ‍warranted on amaranths and commercial crops under controlled greenhouse and field conditions to evaluate the biofertilizer efficacy of these isolates, particularly the consortium. This will be the crucial next step to confirm their potential as candidate biofertilizers before their application as biofertilizers for amaranths.

## Citation

Talamantes-Herrera, F. A., Barrera-Pacheco, A., Espitia-Rangel, E., and Barba-de la Rosa, A. P. (2026) Characterization of Culturable Plant Growth-promoting Bacteria Isolated from *Amaranthus hypochondriacus* L. Roots. *Microbes Environ ***41**: ME26025.

https://doi.org/10.1264/jsme2.ME26025

## Figures and Tables

**Fig. 1.**
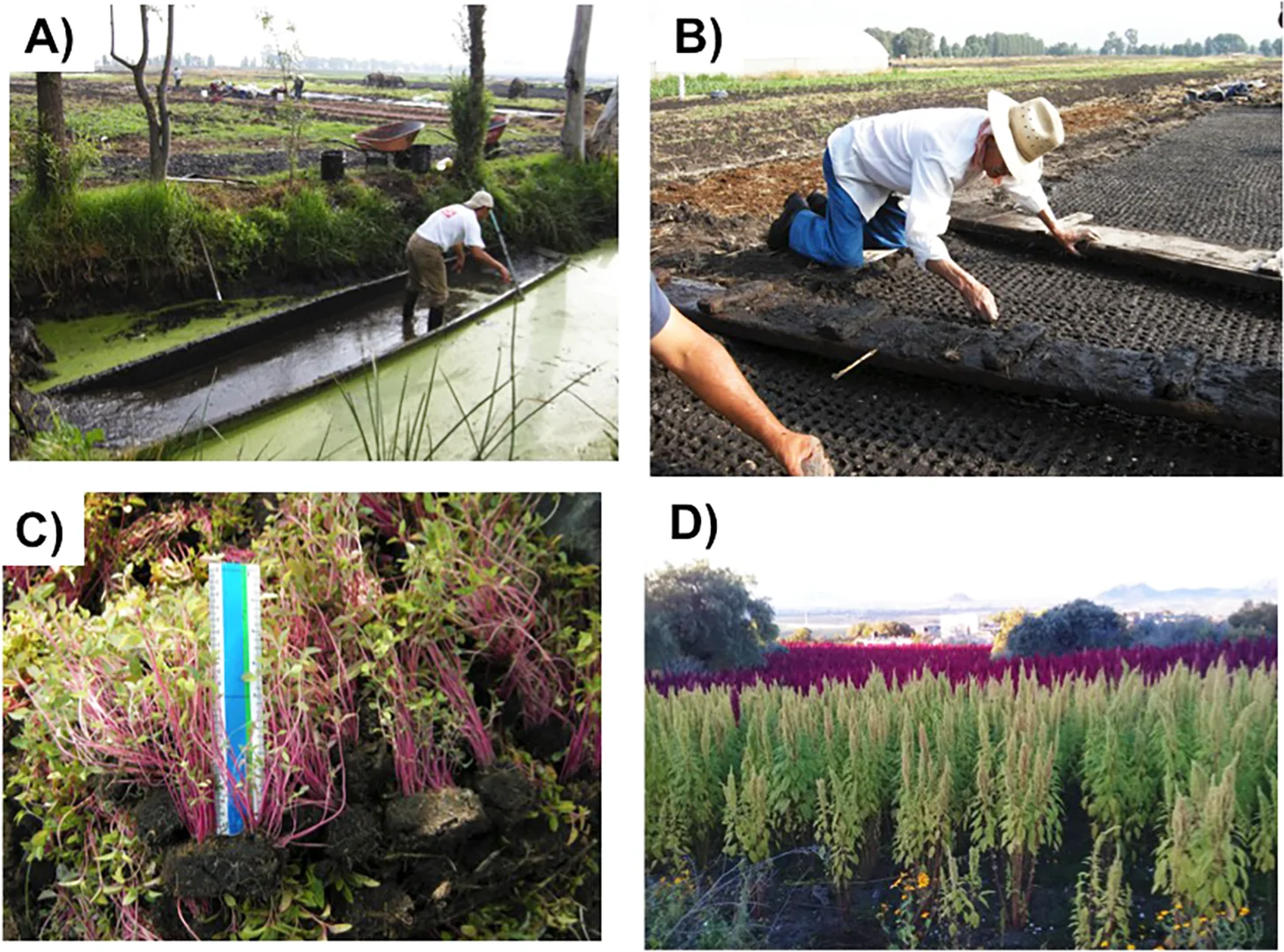
Amaranth cultivation at Xochimilco, Mexico city. A) Soil from rivers collected to form chinampas; B) amaranth seeds are sown in chinampas; C) amaranth seedlings are transplanted to nearby fields; D) mature amaranth plants.

**Fig. 2.**
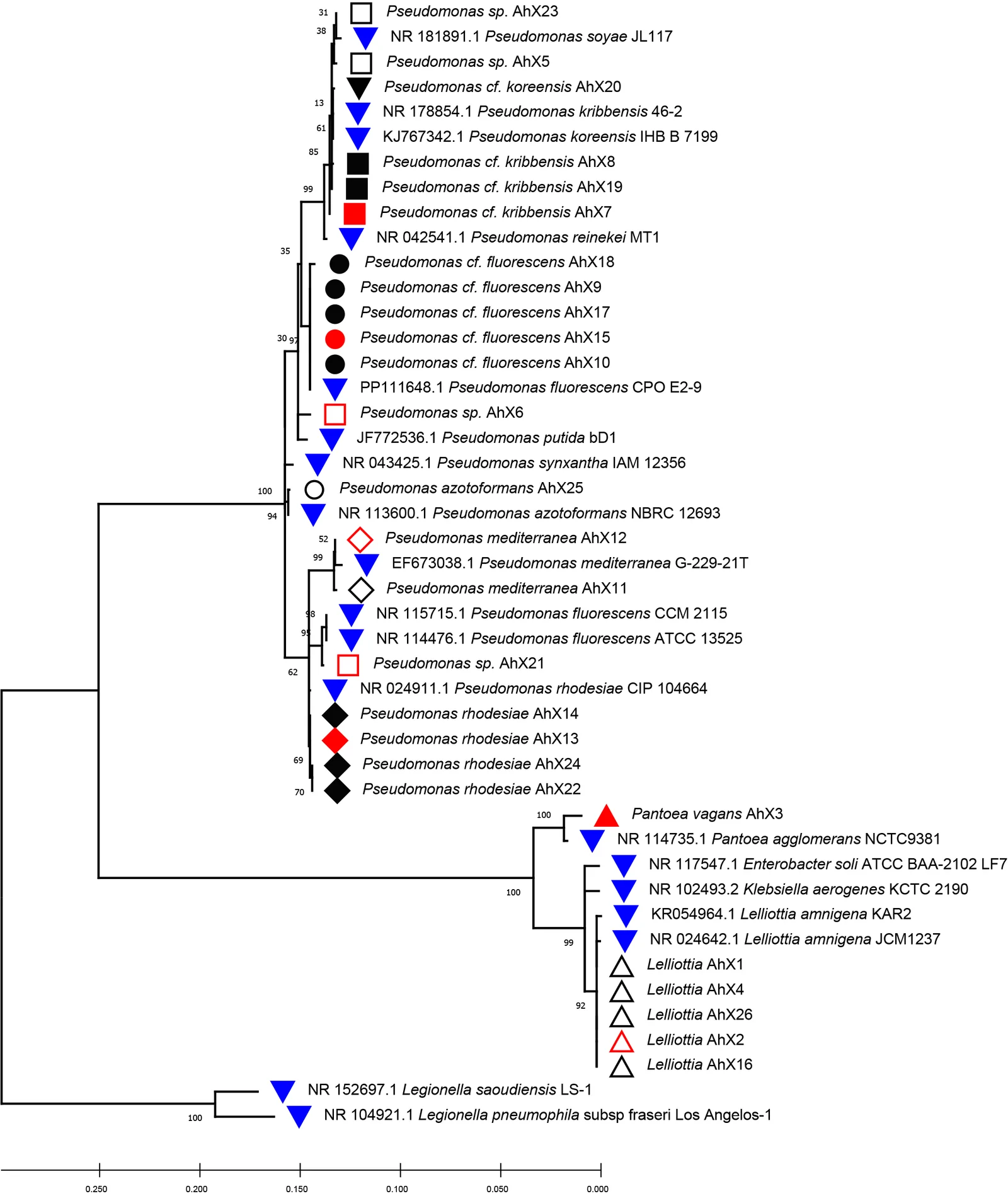
Maximum likelihood phylogenetic tree based on 16S rRNA gene sequences showing relationships of amaranth root endophytes with closely related type strains. The tree was constructed using the HKY+G+I substitution model with 2,000 bootstrap replicates. Bootstrap values (≥50%) are shown at the nodes. Nineteen reference type strain sequences are shown in blue (NCBI accession numbers provided in Methods). *Legionella* sequences (NR_104921.1, NR_152697.1) were used as an outgroup. Isolates selected for the *Arabidopsis* inoculation assay (AhX2, AhX3, AhX13, and AhX15) are shown in red. “*cf.*” (*confer*) indicates a tentative species assignment; “*sp.*” indicates that no confident species-level identification was made.

**Fig. 3.**
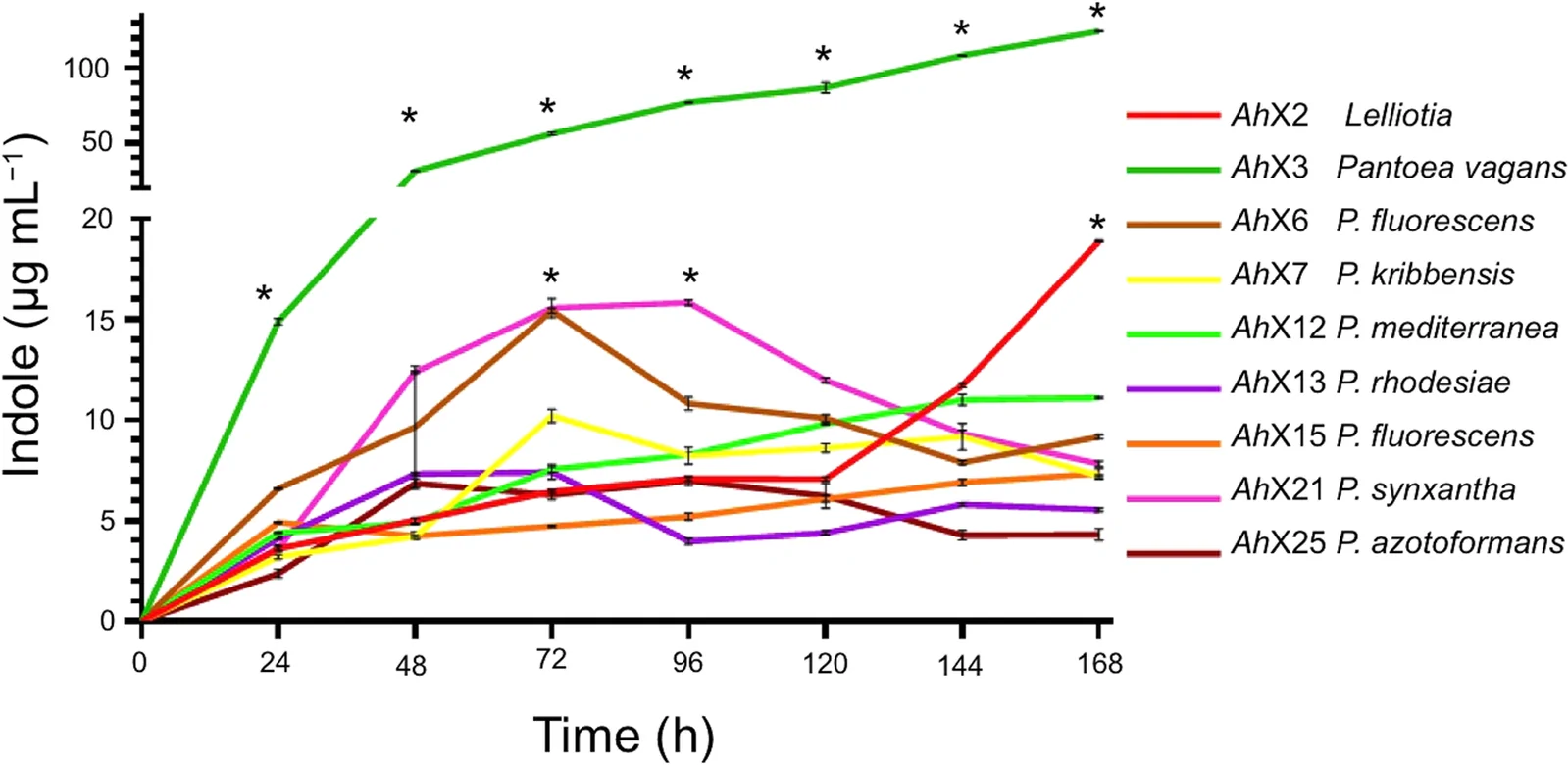
Total indole production by endophytic bacteria isolated from amaranth roots. Indole production was quantified using Salkowski’s reagent. ANOVA and Tukey’s post hoc test were performed. Asterisks show significant differences between means at each time-point at *P*<0.05.

**Fig. 4.**
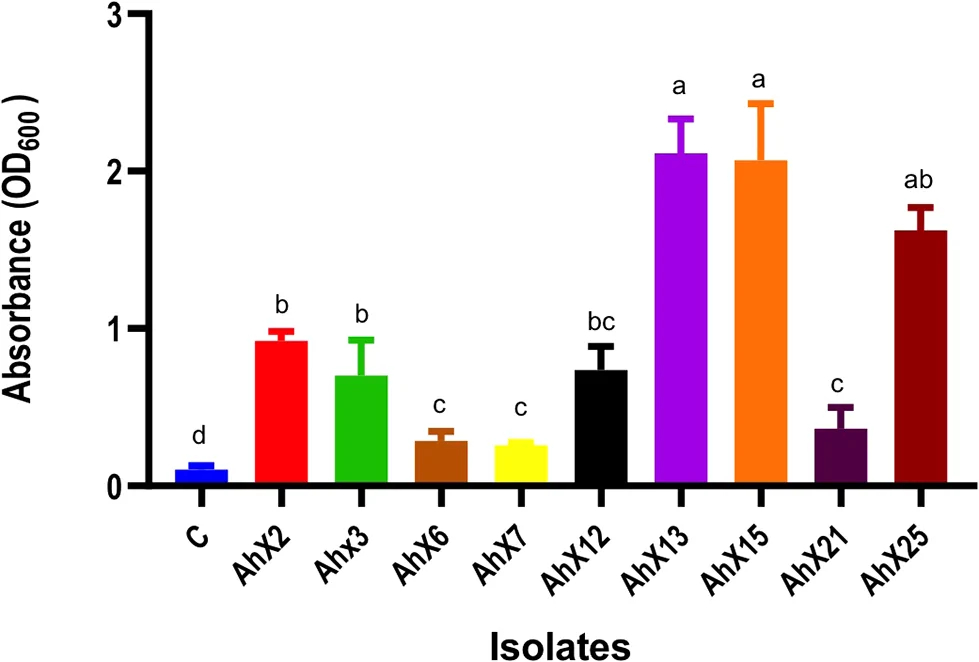
Biofilm production by endophytic bacteria isolated from amaranth roots. Biofilm production was quantified by the crystal violet stain protocol. The Kruskal-Wallis and Dunn’s tests were performed. Data show the mean±standard deviation. Different letters shows significant differences among mean values at *P*<0.05.

**Fig. 5.**
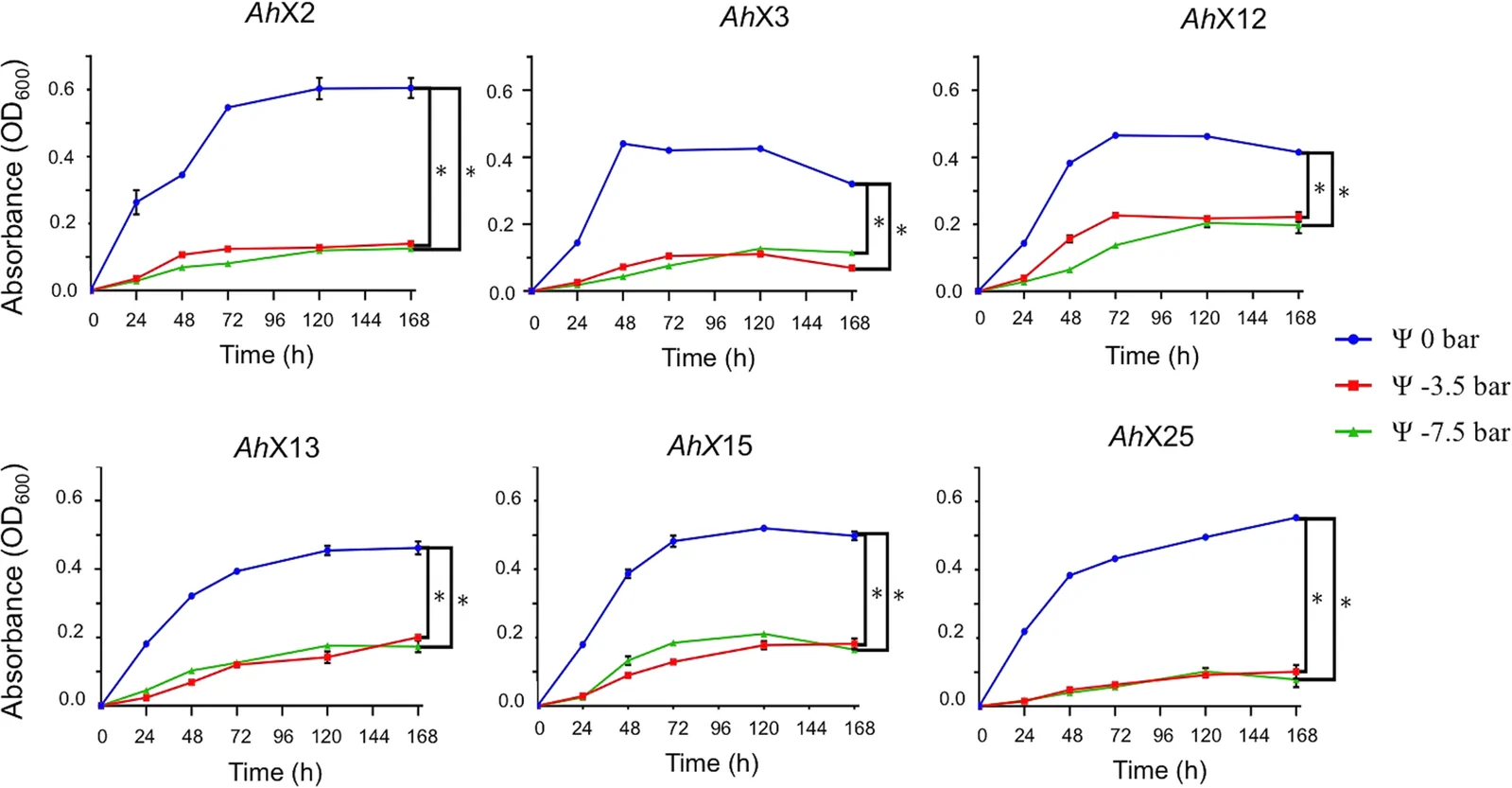
Osmotic stress tolerance of endophytic bacteria isolated from amaranth roots. Bacteria were subjected to two osmotic stress conditions. ANOVA and Tukey’s post hoc test were performed. Asterisks show significant differences among treatments at *P*<0.05.

**Fig. 6.**
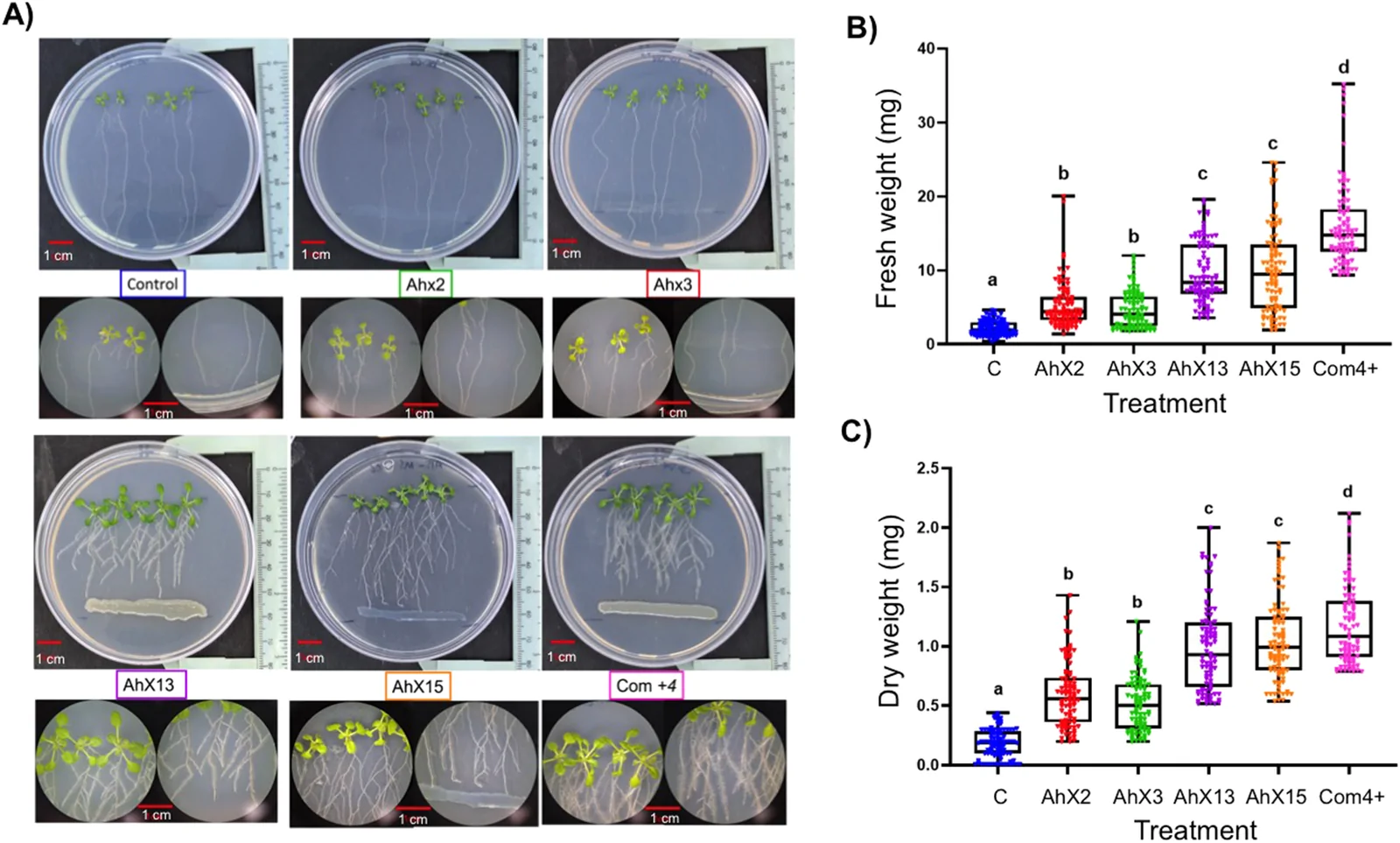
Growth promotion of *Arabidopsis thaliana* Col-0 plantlets by selected amaranth root endophytes. (A) Representative images of 15-day-old plantlets grown on MS agar plates 7 days post-inoculation with AhX2 (*Lelliottia*), AhX3 (*Pantoea vagans*), AhX13 (*Pseudomonas rhodesiae*), AhX15 (*Pseudomonas cf. fluorescens*), and Com4+ (a consortium containing all four isolates) as well as a non-inoculated control. Scale bar=1 cm. (B) Fresh weight and (C) dry weight of plantlets from each treatment. Box plots show the median (line within the box), interquartile range (box bounds), and minimum and maximum values (whiskers). Different letters (a, b, c, d) above the boxes indicate significant differences at *P*<0.05 based on linear mixed-effects models with treatment as the fixed effect and replicate as the random intercept, followed by Tukey-adjusted pairwise comparisons. Treatments sharing the same letter were not significantly different. *n*=15 plants per treatment from one representative experiment, repeated three times with similar results.

**Table 1.** Taxonomic classification of amaranth root endophytes and their iron- and phosphate-solubilizing abilities

**Endophyte ID**	**Taxonomic classification**	**SPI**	**PSI**
*Ah*X1	*Lelliottia*	2.1±0.0^f^	
***Ah*X2**	***Lelliottia***	**2.1±0.0^f^**	**2.4±0.1^g^**
***Ah*X3**	***P. vagans***	**4.1±0.0^b^**	**3.6±0.2^bc^**
*Ah*X4	*Lelliottia*	2.2±0.1^f^	
*Ah*X5	*Pseudomonas sp.*	2.1±0.1^f^	
***Ah*X6**	***Pseudomonas sp.***	**2.2±0.0^f^**	**2.2±0.1^h^**
***Ah*X7**	***P.** cf. **kribbensis***	**2.1±0.0^f^**	**2.3±0.1^gh^**
*Ah*X8	*P. cf. kribbensis*	2.1±0.0^f^	
*Ah*X9	*P. cf. fluorescens*		3.5±0.0^bc^
*Ah*X10	*P. cf. fluorescens*		3.4±0.2^c^
*Ah*X11	*P. mediterranea*		2.4±0.0^g^
***Ah*X12**	***P. mediterranea***	**5.7±0.1^a^**	**2.5±0.0^g^**
***Ah*X13**	***P. rhodesiae***	**2.9±0.2^d^**	**3.9±0.1^b^**
*Ah*X14	*P. rhodesiae*		4.5±0.0^a^
***Ah*X15**	***P.** **cf. fluorescens***	**2.4±0.1^e^**	**3.8±0.2^b^**
*Ah*X16	*Lelliottia*	2.2±0.1^f^	
*Ah*X17	*P. cf. fluorescens*		3.1±0.1^d^
*Ah*X18	*P. cf. fluorescens*		3.3±0.2^cd^
*Ah*X19	*P. cf. kribbensis*		2.3±0.1^gh^
***Ah*X20**	***P. cf. koreensis***	**2.1±0.0^f^**	**2.1±0.1^h^**
***Ah*X21**	***Pseudomonas sp.***	**2.1±0.0^f^**	**3.5±0.0^bc^**
*Ah*X22	*P. rhodesiae*		3.9±0.1^b^
*Ah*X23	*Pseudomonas sp.*	2.2±0.0^f^	
*Ah*X24	*P. rhodesiae*		3.6±0.2^b^
***Ah*X25**	***P. azotoformans***	**3.5±0.1^c^**	**3.0±0.2^df^**
*Ah*X26	*Lelliottia*	2.1±0.0^f^	

SPI, siderophore production index; PSI, phosphate-solubilizing index. *Ah*=*Amaranthus hypochondriacus*, X=Xochimilco, # of Isolate; The Kruskal-Wallis and Dunn’s tests were performed. Data are the mean±SD of triplicates. The same letters in a column were not significantly different at *P*<0.05. Bold letters indicate isolates that were selected for further study.
